# An Imagery-Based Road Map to Tackle Maladaptive Motivation in Clinical Disorders

**DOI:** 10.3389/fpsyt.2015.00014

**Published:** 2015-02-12

**Authors:** Jon May, Jackie Andrade, David J. Kavanagh

**Affiliations:** ^1^School of Psychology, Cognition Institute, Plymouth University, Plymouth, UK; ^2^Institute of Health and Biomedical Innovation and School of Psychology and Counselling, Queensland University of Technology, Brisbane, QLD, Australia

**Keywords:** transdiagnostic, therapy, treatment, cognitive, motivational, goals, addiction, craving

## Introduction

There is now a widespread recognition of the importance of mental imagery in a range of clinical disorders (1). This provides the potential for a transdiagnostic route to integrate some aspects of these disorders and their treatment within a common framework. This opinion piece argues that we need to understand why imagery is such a central and recurring feature, if we are to progress theories of the origin and maintenance of disorders. This will aid us in identifying therapeutic techniques that are not simply targeting imagery as a symptom, but as a manifestation of an underlying problem.

As papers in this issue highlight, imagery is a central feature across many clinical disorders, but has been ascribed varying roles. For example, the involuntary occurrence of traumatic memories is a diagnostic criterion for PTSD (2), and it has been suggested that multisensory imagery of traumatic events normally serves a functional role in allowing the individual to reappraise the situation (3), but that this re-appraisal is disabled by extreme affective responses. In contrast to the disabling flashbacks associated with PTSD, depressed adults who experience suicidal ideation often report “flash forward” imagery related to suicidal acts (4), motivating them to self-harm. Socially anxious individuals who engage in visual imagery about giving a talk in public become more anxious and make more negative predictions about future performance than others who engage in more abstract, semantic processing of the past event (5). People with Obsessive Compulsive Disorder (OCD) frequently report imagery of past adverse events, and imagery seems to be associated with severity (6). The content of intrusive imagery has been related to psychotic symptoms (7), including visual images of the catastrophic fears associated with paranoia and persecution. Imagery has been argued (8) to play a role in the maintenance of psychosis through negative appraisals of imagined voices, misattribution of sensations to external sources, by the induction of negative mood states that trigger voices, and through maintenance of negative schemas. In addiction and substance dependence, Elaborated Intrusion (EI) Theory (9, 10) emphasizes the causal role that imagery plays in substance use, through its role in motivating an individual to pursue goals directed toward achieving the pleasurable outcomes associated with substance use.

In this opinion piece, we would like to put forward the proposition that the motivating role that imagery plays in behavior may be a key reason for its presence in this wide range of psychopathologies, and that understanding its motivating role can help us identify common features in disorders and their treatments. It has already been recognized that the link between imagery and affect gives imagery a causative role in affective disorders (11), with the emotional amplifier hypothesis suggesting that in bipolar and anxiety disorders, state-congruent imagery leads to further affective responses, in a vicious cycle. Within EI Theory, the affective consequences of imagery are also used to explain why a cycle of elaboration follows an initial intrusive thought. The intrusive thoughts themselves are seen as the product of associative processes relating to both environmental cues and cognitions. Previous work on EI Theory focused initially on desires, and then on motivation in general. Our suggestion here moves a step further, highlighting its potential relevance to dysfunctional motivational imagery in multiple disorders.

## Elaborated Intrusion Theory

Elaborated Intrusion Theory was initially developed to provide a cognitive account of processes involved in the maintenance of substance dependence, and to demonstrate that dependence for different substances shared a common functional basis, despite gross differences in their psychopharmacology. More provocatively, EI theory saw substance-related craving as just an extreme example of a continuum of desire, and argued that it recruited the normal cognitive processes involved in everyday desires. Since its publication ten years ago, the key premises of EI Theory and hypotheses drawn from it have been supported in a substantial body of research (10, 12–14).

According to EI Theory, environmental cues, physiological symptoms (i.e., withdrawal or deficit states), affect, and automatic or overlearned associations that operate outside of awareness do not constitute desires, because the individual is not aware of them. Instead, they generate cognitive activity that, depending upon the task context and other cognitive activity, can become sufficiently strong to become the focus of attention, and be subjectively experienced as an apparently spontaneous thought. When this thought is about a target that is strongly associated with pleasure or relief, it is highly salient, and generates a cascade of cognitive activity, which draws on further associations to elaborate the thought. Sensory imagery is often involved in the intrusion and is a key component of the elaboration, leading to anticipatory responses and augmenting its positive affective impact. However, the sensory focus also elicits an increased awareness of current deficit states, which is increasingly aversive as acquisition of the target is impeded. If the person is trying to withstand the temptation, they may also experience guilt about having the thought, or worry that it means they are losing control. While the affective consequences of the imagery lead to its continued elaboration and perpetuate the craving episode, its functional role is to motivate behavior toward satisfying the desire: it sets up a behavioral goal that competes with and can displace current goals.

## Targeting Imagery Components

Conceptualizing desire in this way has allowed us to devise therapeutic and behavior change techniques that target the two key components of desire: the initial intrusion and its subsequent elaboration. As the intrusion follows automatic processing of triggers external and internal to the individual, the frequency of intrusions can be reduced by either reducing those triggers or impairing their automated processing. Many existing approaches can be seen to operate through their effect upon intrusions: medications that ameliorate withdrawal symptoms are an obvious way of reducing physiological cues; mood enhancement can target the contribution of negative affect; and attentional retraining can lower an individual’s preconscious processing of environmental cues associated with substance use. Once an intrusion has occurred, training in acceptance and a non-judgmental response style can help in letting the thought fade away and be displaced in focal awareness by mindful meditation or other cognitive tasks, without its elaboration dominating thought. If the thought does remain in focal awareness, a variety of imagery-based attentional control tasks can be employed to compete for imagery resources, blocking the sensory imagery of the target that maintains the elaborative cycle, shortening the duration, and weakening the affective intensity of the craving episode (12).

There are clear parallels here with the ways in which imagery is being addressed within therapeutic developments in clinical psychology. For example, imagery rescripting (15) was initially developed to help people with PTSD whose high levels of shame or guilt prevented them mentally reliving experiences, by reframing the event from the perspective of a neutral observer, weakening the emotional response. Different resolutions for the event could then be reimagined, resolving the guilt. This allows different patterns of elaboration to follow any intrusive thoughts, replacing the negative emotions associated with the original elaborations. It has since been applied to anxiety (16), eating disorders (17), and psychosis (8). Imagery-based cognitive therapy can help individuals to make the imagery more realistic (18). Within EI Theory, rescripting can be seen as an alternate elaborative strategy to a concurrent task or a modification of affective responses using mindfulness.

## Imagery Supports Motivation and Goals

Desire does not only lead us to pursue illicit or unhealthy targets, of course: the strength of hockey players’ cravings to play hockey is also determined by the vividness of the sensory imagery that they experience during the episode (19). Imagery-based desires are part of normal motivational processes, directing us toward long-term as well as short-term goals, by bridging the gap in time, and help us deal with challenges in goal acquisition. They support a wide range of behaviors beyond consumption, and EI Theory can be applied to enhance goals for positive behaviors, as well as helping people abstain from dysfunctional ones. In fact, a modification of motivational interviewing called Functional Imagery Training has been developed (20), where people are encouraged to form mental images of the incentives for change, the path toward that change, and their past successes in attaining similar goals. Providing a rich repertoire of vivid positive imagery associated with attainment of an otherwise abstract and general long-term goal allows that goal to compete strongly in the elaborative cycle with the pleasurable sensory images of conflicting short-term goals, which are often highly concrete and specific. By strengthening thoughts of succeeding in behavior change, the very cues that otherwise lead to failure can be recruited to enhance success, in a positive feedback loop, with small steps toward the goal increasing the likelihood of subsequent steps being taken.

Motivation and goal fulfillment in behavior change may seem a long way from the issue of imagery within clinical disorders, but it is worth considering whether the experience of vivid mental images within psychopathologies might not also be linked to disorders of goal formation (21). Holmes et al. (11) raise this possibility in relation to bipolar disorder, arguing that vivid, positive imagery can foster creativity during hypomania but leads to pursuit of unrealistic goals during mania. The goal follows the image, as in their example of someone whose vivid image of a red sports car might prompt them to visit a car showroom. We hypothesize that the relationship between images and goals is broader, so images strengthen pursuit of existing goals as well as stimulating goal formation, and those existing goals may be goals for immediate safety or comfort rather than longer term well-being. In anxiety disorders, imagery of negative and threatening consequences sustains goals of staying safe or avoiding harm. Perhaps this can be taken further, and the flashbacks within PTSD that have been suggested as supporting re-appraisal (3) can be interpreted as part of a general problem-solving process where the individual seeks to imagine ways that they could have behaved in the traumatic situation, which could be recruited in future situations. Similarly, suicidal imagery may motivate goal directed behaviors through increasing the cognitive availability of the actions as solutions to the individual’s distress (22) or it may motivate more constructive ways of achieving the imagined consequences of the actions (4). The common feature across these interpretations of imagery is that the intrusion has been triggered because an unresolved and emotionally salient problem remains unsolved. Intrusive imagery is our cognitive mechanism’s way of prioritizing an unsolved problem, just as other goals stay active and intrusion-prone until fulfilled (23, 24) – but if the problem cannot be resolved, the imagery becomes maladaptive, and by sustaining a dysfunctional or unachievable goal leads to an affective disorder. In depression, for example, negative self-related ruminations displace ideas of success and lead to a cycle of anhedonia and loss of agency, with the individual finding it near impossible to imagine any future goals.

## Road Map to Tackle Maladaptive Imagery

We suggest that EI theory could provide a road map for effectively tackling imagery of maladaptive goals across clinical disorders: helping to identify risky situations for temptation and loss of control; reducing attention to intrusive thoughts through mindfulness or acceptance training; reducing the elaboration of dysfunctional desires through modality-specific interference [e.g., concurrent visuospatial tasks while recalling trauma memories (25)], or rescripting the content of the image (e.g., adjusting overly positive expectancies of substance use). Treatments targeting imagery should also consider the need to address the goals that those images support, identifying alternative, adaptive goals, and training alternative imagery to boost desire for those goals and rehearse pathways toward them, as in Functional Imagery Training (20).

This interpretation of imagery research within clinical psychology is speculative, and glosses over details and differences between the particular ways in which imagery may be critical to particular disorders. Further argument and empirical work are needed to explore the value of linking clinical conceptualizations of imagery to the rapidly developing work in cognitive psychology and behavior change. Imagery of course has a long history in clinical psychology, from systematic desensitization to covert practice, and has been extensively used to enhance sporting performance: the conceptualization of its role and application in the current paper needs to recognize and accommodate these phenomena, as well as expanding its explanatory power. Reappraising imagery as one part of a larger set of processes that act in consort to guide people in their everyday behavior raises the possibility of engaging clinically with each part of that mechanism, understanding why and how imagery is being distorted or populated by maladaptive content in different ways in a range of disorders. Understanding even part of the relationship would, we believe, justify the effort.

## Conflict of Interest Statement

The authors declare that the research was conducted in the absence of any commercial or financial relationships that could be construed as a potential conflict of interest.
